# Comparative plastome analysis of *Blumea*, with implications for genome evolution and phylogeny of Asteroideae

**DOI:** 10.1002/ece3.7614

**Published:** 2021-05-06

**Authors:** Abdullah, Furrukh Mehmood, Abdur Rahim, Parviz Heidari, Ibrar Ahmed, Péter Poczai

**Affiliations:** ^1^ Department of Biochemistry Faculty of Biological Sciences Quaid‐i‐Azam University Islamabad Pakistan; ^2^ Department of Zoology Post Graduate College Nowshera Abdul Wali Khan University Mardan Pakistan; ^3^ Faculty of Agriculture Shahrood University of Technology Shahrood Iran; ^4^ Alpha Genomics Private Limited Islamabad Pakistan; ^5^ Finnish Museum of Natural History University of Helsinki Helsinki Finland; ^6^ Faculty of Biological and Environmental Sciences University of Helsinki Helsinki Finland

**Keywords:** Asteraceae, Asteroideae, barcode, *Blumea*, oligonucleotide repeat, plastome, polymorphic loci

## Abstract

The genus *Blumea* (Asteroideae, Asteraceae) comprises about 100 species, including herbs, shrubs, and small trees. Previous studies have been unable to resolve taxonomic issues and the phylogeny of the genus *Blumea* due to the low polymorphism of molecular markers. Therefore, suitable polymorphic regions need to be identified. Here, we de novo assembled plastomes of the three *Blumea* species *B*. *oxyodonta*, *B. tenella*, and *B. balsamifera* and compared them with 26 other species of Asteroideae after correction of annotations. These species have quadripartite plastomes with similar gene content, genome organization, and inverted repeat contraction and expansion comprising 113 genes, including 80 protein‐coding, 29 transfer RNA, and 4 ribosomal RNA genes. The comparative analysis of codon usage, amino acid frequency, microsatellite repeats, oligonucleotide repeats, and transition and transversion substitutions has revealed high resemblance among the newly assembled species of *Blumea*. We identified 10 highly polymorphic regions with nucleotide diversity above 0.02, including *rps*16‐*trn*Q, *ycf*1, *ndh*F‐*rpl*32, *pet*N‐*psb*M, and *rpl*32‐*trn*L, and they may be suitable for the development of robust, authentic, and cost‐effective markers for barcoding and inference of the phylogeny of the genus *Blumea*. Among these highly polymorphic regions, five regions also co‐occurred with oligonucleotide repeats and support use of repeats as a proxy for the identification of polymorphic loci. The phylogenetic analysis revealed a close relationship between *Blumea* and *Pluchea* within the tribe Inuleae. At tribe level, our phylogeny supports a sister relationship between Astereae and Anthemideae rooted as Gnaphalieae, Calenduleae, and Senecioneae. These results are contradictory to recent studies which reported a sister relationship between “Senecioneae and Anthemideae” and “Astereae and Gnaphalieae” or a sister relationship between Astereae and Gnaphalieae rooted as Calenduleae, Anthemideae, and then Senecioneae using nuclear genome sequences. The conflicting phylogenetic signals observed at the tribal level between plastidt and nuclear genome data require further investigation.

## Graphical abstract

Our study provides insight into plastome structure evolution of the genus *Blumea* and the subfamily Asteroideae (Asteraceae). Previously, the phylogeny of *Blumea* could not be resolved due to lack of knowledge of suitable polymorphic loci. Based on comparative genomics, we report suitable polymorphic loci which are also co‐occur with repeats and support the role of repeats as proxy for identification of polymorphic loci. The conflicting phylogenetic signals observed at tribe level between plastome and nuclear genome data may be due to reticulate and abrupt evolution which require further investigation.

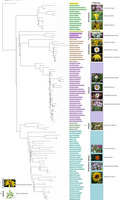

## 1 INTRODUCTION

The plastome has a mostly quadripartite structure in which a pair of inverted repeats (IRa and IRb) separate large single‐copy (LSC) and small single‐copy (SSC) regions (Daniell et al., 2016; Henriquez et al., 2020a, 2020b). However, a mixture of circular and linear plastomes is also reported (Oldenburg & Bendich, 2016). The plastome shows paternal inheritance in some gymnosperms (Neale & Sederoff, 1989) and maternal inheritance in most angiosperms (Daniell, 2007). The reported size of the plastome ranged from 107 kb (*Cathaya argyrophylla* Chun & Kaung) to 218 kb (*Pelargonium × hortorum* L.H. Bailey) (Daniell et al., 2016). Several types of mutations occur within the plastome, including single‐nucleotide polymorphism (SNPs), insertions/deletions (indels), and inversions (Henriquez et al., 2020b; Wang et al., 2015; Xu et al., 2015). Certain regions of the plastome are more predisposed to mutations than others and show a differential evolution rate (Abdullah et al., 2019; Abdullah, Waseem, et al., 2020; Iram et al., 2019). Polymorphism of the plastome is suitable for investigating population genetics, phylogenetics, and barcoding of plants (Ahmed, 2014; Mehmood, Abdullah, Shahzadi, et al., 2020; Mehmood, Abdullah, Ubaid, et al., 2020; Shahzadi et al., 2020; Teshome et al., 2020). The plastome has a uniparental inheritance and appropriate polymorphism (Palmer, 1985). These properties make polymorphism of the plastome a suitable molecular marker for resolving taxonomic issues and infer the phylogeny of plants with high resolution (Daniell et al., 2016). Moreover, the plastome markers can be robust, authentic, and cost‐effective (Ahmed et al., 2013; Nguyen et al., 2018). Therefore, several studies have focused on the identification of suitable polymorphic loci to resolve the taxonomic discrepancies of various plant lineages (Abdullah, Mehmood, et al., 2020; Iram et al., 2019; Shahzadi et al., 2020; Yang et al., 2019). Comparative analyses of whole plastomes not only provide in‐depth insight into the evolution of the plastome but are also helpful in the identification of polymorphic loci (Abdullah, Henriquez, Mehmood, Shahzadi, et al., 2020; Abdullah, Mehmood, et al., 2020; Henriquez et al., 2020b).

The family Asteraceae is among three megadiverse families. The number of estimated species of the family range from 25,000 to 35,000, and they comprise up to 10% of all the flowering plant species (Mandel et al., 2019). Species of the family Asteraceae exist in every type of habitat and every continent including Antarctica (Barreda et al., 2015; Mandel et al., 2019). This family is divided into 13 subfamilies (Mandel et al., 2019; Panero & Crozier, 2016; Panero et al., 2014). Asteroideae (Cass.) Lindl. comprises 17,000+ species, which makes it the largest subfamily in Asteraceae (Mandel et al., 2019). Asteroideae is the recently diverged, ~37 mya (Mandel et al., 2019) or ~45 mya (Panero & Crozier, 2016), subfamily of Asteraceae and is divided into three super tribes, including Helianthodae (6,611 species), Senecionadae (3,480), and Asterodae (7,109) (Panero & Crozier, 2016). These are further divided into many tribes, of which the main tribes are the Heliantheae alliance (∼5,600 species), Senecioneae (3,500), Astereae (3,080), Anthemideae (1,800), Gnaphalieae (1,240), Inuleae (687), and Calenduleae (120) (Mandel et al., 2019; Watson et al., 2020). The species of Asteroideae are also very diverse in distribution, similar to the family Asteraceae, and are distributed in America, Asia, Africa, Europe, Oceania, and the Pacific Island region (Mandel et al., 2019; Panero & Crozier, 2016). Watson et al. (2020) referred to the five tribes Senecioneae, Astereae, Anthemideae, Gnaphalieae, and Calenduleae as the Fab(ulous) Five. They are taxonomically difficult tribes, and conflicting phylogenetic signals were recorded for these tribes based on the plastid, nuclear, and transcriptomic data (for details see the discussion) (Fu et al., 2016; Mandel et al., 2019; Panero & Crozier, 2016; Panero et al., 2014; Watson et al., 2020).

The genus *Blumea* DC. belongs to the tribe Inuleae of the subfamily Asteroideae (Pornpongrungrueng et al., 2016). This genus contains 100 species of herbs, shrubs, and small trees (Pornpongrungrueng et al., 2009). The species are distributed throughout the Old World tropics (Pornpongrungrueng et al., 2009) and are most diverse in Australia, Africa, and Asia (Pornpongrungrueng et al., 2016). Members of the genus have ecological and economic value (Zhang et al., 2019). Most of the species of the genus *Blumea* are enriched with flavonoids, terpenes, acetylenic thiophenes, triterpenoids, sterols, and essential oils (Chen et al., 2009; Pang et al., 2014). The species of *Blumea* have shown several medicinal activities, including anticancer, antioxidant, antimicrobial, anti‐inflammatory, antiplasmodial, antityrosinase, and anti‐obesity effects (Chen et al., 2009; Pang et al., 2014). Hepatoprotective, platelet aggregation, percutaneous penetration, wound healing, and superoxide radical scavenger properties are also reported (Chen et al., 2009; Pang et al., 2014). *Blumea balsamifera* (L.) DC. is considered to be one of the most important medicinal species (Pang et al., 2014).

The genus *Blumea* is monophyletic if the genera *Blumeopsis* Gagnep. and *Merrittia* Merr. are included (Pornpongrungrueng et al., 2007, 2009). The genus is divided into three clades by inferring phylogeny using plastid markers (*trn*L‐F and *trn*H‐*psb*A), the nuclear ribosomal internal transcribed spacer (ITS) region, and the 5S‐NTS (nontranscribed spacer) (Pornpongrungrueng et al., 2009; Zhang et al., 2019). Certain discrepancies still exist at the intra‐genus level, and several relationships are unresolved at the species level (Chen et al., 2009). Low bootstrapping support was observed for various nodes. Therefore, researchers have suggested identifying suitable polymorphic loci to elucidate the phylogenetic relationships (Pornpongrungrueng et al., 2007).

Here, we are interested in (a) providing new insight into the plastome of the genus *Blumea* and performing comparative plastid genomics with other species of the subfamily Asteroideae; (b) reconstructing the phylogeny within the subfamily Asteroideae; (c) identifying suitable polymorphic loci for the phylogenetic inference of the genus *Blumea*; (d) elucidating the role of repeats as a proxy for the identification of mutational hot spots.

## 2 MATERIALS AND METHODS

### 2.1 Genome assembly and coverage depth analysis

We downloaded the short reads of all three *Blumea* species from the Sequence Read Archive (SRA) of the National Center for Biotechnology Information (NCBI) available under project numbers PRJNA438407 and PRJNA522689. The whole genome shotgun data of *B*. *oxyodonta* and *B. balsamifera* were generated using BGISEQ‐500 with short reads of 100 bp from a single end (Liu et al., 2019), whereas the genome of *B. tenella* was sequenced using Illumina True‐Seq with short reads of 250 bp. Data accession numbers and quantity are presented in Table 1.

**TABLE 1:** Accession numbers, quantity of raw data, and coverage depth of de novo assembled plastomes

Species	SRA accession no.	Data downloaded (GB)	Coverage depth	WGS reads (millions)	Plastome reads (millions)	GenBank submission
*Blumea oxyodonta*	SRR7121564	1.89	98×	3.38	0.15	BK013128
*Blumea tenella*	SRR8666706	1.69	119×	2.83	0.15	BK013129
*Blumea balsamifera*	SRR7191154	1.72	130×	3.07	0.20	BK013127

Abbreviation: SRA, Sequence Read Archive; WGS, whole genome sequencing.

To assemble the plastome, we first used the BWA‐MEM algorithm with default parameters (Li & Durbin, 2009) and mapped all reads for each species to *Aster hersileoides* C.K. Schneid., *Tagetes erecta* L., *Ambrosia trifida* L., *Bidens torta* Sherff, and *Artemisia ordosica* Krasch. This approach was used to avoid contamination of the nuclear and mitochondrial origin reads. The plastomes were de novo assembled by Velvet v.1.2.10 (Zerbino & Birney, 2008) integrated in Geneious R8.1 (Kearse et al., 2012) using extracted reads. All generated contigs of Velvet were combined in a specific order using the de novo assembly option of Geneious R8.1 (Kearse et al., 2012). This helped us to assemble the plastome of the *Blumea* species in a single contig from total contigs generated by Velvet. The single contig of the *Blumea* species was assembled from 5–10 long contigs without any gap. The LSC, IR, and SSC regions were defined based on the manual inspection of scaffolds. The integrity of the assembled plastome was validated, and coverage depth analysis was performed by mapping the original reads of each genome to their respective genome using BWA‐MEM (Li & Durbin, 2009). We did not predict any gap in the assembled plastome, which further confirmed the high quality of the assembled plastome. However, through mapping of short reads we avoid some nucleotide mismatches in the genomes.

Plastomes were annotated using GeSeq (Tillich et al., 2017), whereas transfer RNAs (tRNAs) were confirmed based on ARAGORN v.1.2.38 (Laslett & Canback, 2004) and tRNAscan‐SE v.2.0.5 (Chan & Lowe, 2019). The annotations of protein‐coding genes were further confirmed by comparison with other homologue genes in the public database. The five‐column tab‐delimited file was generated from annotated genomes using GB2Sequin (Lehwark & Greiner, 2019) and was submitted to NCBI GenBank under specific accession numbers (Table 1). The circular map of the *Blumea* plastome was drawn using Chloroplot to show genome organization and gene content (Zheng et al., 2020).

### 2.2 Reannotations of plastomes and comparative genomics among the species of the subfamily Asteroideae

For comparative genomics among species of the subfamily Asteroideae with the species of the genus *Blumea*, we retrieved 26 other species of the subfamily Asteroideae from the NCBI (Table 2). These species were selected to cover all the main tribes of Asteroideae, including Anthemideae, Astereae, Gnaphalieae, the Heliantheae alliance, and Senecioneae, whereas the species of *Blumea* cover the tribe Inuleae. For a good comparison, we first reannotated all the 26 species using an approach similar to that used for the annotation of *Blumea* species as previous studies showed certain errors exist in the annotation of the plastome available in a public database (Amiryousefi et al., 2018a). After that, we compared genome features including the size of the complete plastome, LSC, SSC, and IR along with the gene and intron content. The arrangement of genes in the genomes was determined using Mauve (Darling et al., 2004), whereas contraction and expansion of IRs were visualized using IRscope (Amiryousefi et al., 2018b).

**TABLE 2:** Comparison of plastomes of 29 species of the subfamily Asteroideae with *Blumea* species

Species	Genome length (bp)				GC content (%)				Accession no.
	Total	LSC	SSC	IR	Total	LSC	SSC	IR	
*Blumea balsamifera*	151,176	82,746	18,466	24,982	37.6	35.8	31.1	43	BK013127
*Blumea oxyodonta*	150,997	82,745	18,438	24,907	37.5	35.7	30.9	43	BK013128
*Blumea tenella*	150,829	82,478	18,439	24,956	37.6	35.7	30.9	43	BK013129
*Ambrosia trifida*	152,040	83,966	17,894	25,090	37.6	35.7	31.5	43.1	MG029118
*Artemisia ordosica*	151,209	82,980	18,303	24,963	37.4	35.5	30.7	43.1	NC_046571
*Aster hersileoides*	152,345	84,124	18,243	24,989	37.3	35.2	31.2	43.0	MK290823
*Bidens torta*	151,807	84,013	18,443	24,689	37.5	35.5	31.0	43.2	NC_047275
*Chromolaena odorata*	151,270	82,663	18,447	25,080	37.5	35.6	30.8	43.0	NC_050055
*Crossostephium chinense*	151,097	82,819	18,328	24,975	37.4	35.5	30.7	43.1	NC_042725
*Dendrosenecio cheranganiensis*	150,626	83,476	17,768	24,691	37.4	35.6	30.9	43.0	MG560046
*Eclipta alba*	151,733	83,300	18,283	25,075	37.5	35.7	30.7	43.0	MF993496
*Erigeron breviscapus*	152,357	84,871	18,102	24,692	37.1	35.0	31.0	43.1	MK279916
*Flaveria bidentis*	152,230	83,798	18,362	25,035	37.7	35.8	31.4	43.1	MK836182
*Galinsoga parviflora*	151,811	83,594	18,141	25,038	37.7	35.8	31.3	43.1	MK737938
*Helianthus annuus*	151,117	83,536	18,319	24,631	37.6	35.7	31.3	43.2	MK341451
*Heteroplexis incana*	152,605	84,427	18,270	24,954	37.3	35.2	31.3	43.1	NC_048508
*Ismelia carinata*	149,752	82,290	18,416	24,523	37.5	35.6	30.8	43.1	MG710387
*Leucanthemum virgatum*	150,120	82,641	18,435	24,522	37.5	35.6	30.8	43.1	NC_047461
*Ligularia mongolica*	151,118	83,244	18,214	24,830	37.5	35.6	30.7	43.0	NC_039384
*Marshallia obovata*	152,553	83,817	17,910	25,413	37.3	35.4	30.8	42.7	MH037169
*Opisthopappus taihangensis*	151,089	82,877	18,304	24,954	37.5	35.6	30.8	43.1	NC_042787
*Parthenium hysterophorus*	151,912	83,604	18,122	25,093	37.6	35.7	31.4	43.1	MT576959
*Senecio vulgaris*	150,806	82,950	18,214	24,821	37.3	35.4	30.4	42.9	MK654722
*Sphagneticola calendulacea*	151,748	83,270	18,348	25,065	37.5	35.7	30.7	43.0	NC_039346
*Symphyotrichum subulatum*	153,318	85,238	18,226	24,927	37.0	34.8	31.0	43.0	MN541093
*Tagetes erecta*	152,055	83,815	18,065	25,048	37.4	35.4	30.9	43.0	MN203535
*Tithonia diversifolia*	151,161	83,615	18,264	24,641	37.7	35.8	31.4	43.2	MT576958
*Xanthium sibiricum*	151,897	83,846	17,900	25,070	37.5	35.5	31.4	43.0	NC_042232
*Anaphalis sinica*	152,718	84,546	18,488	24,842	37.5	35.0	30.8	43.1	KX148081

Abbreviations: IR, inverted repeat; LSC, large single copy; SSC, small single copy.

### 2.3 Comparison of codon usage, amino acid frequency, microsatellites, and oligonucleotide repeats among the species of *Blumea*

The amino acid frequency and relative synonymous codon usage (RSCU) were determined among the *Blumea* species using Geneious R8.1. Microsatellite repeats were determined in *Blumea* plastomes using MISA‐web (Beier et al., 2017) with the following parameters: ≥10 for mononucleotide, ≥5 for dinucleotide, ≥4 for trinucleotide, and ≥3 for tetra‐, penta‐, and hexanucleotide repeats. REPuter (Kurtz et al., 2001) was used to determine oligonucleotide repeats with a minimum size of ≥30 bp and at least 90% similarity. The maximum repeat count was set to 500.

### 2.4 Identification of polymorphic loci in the genus *Blumea*

Multiple alignment was formed among plastomes of all the *Blumea* species, after removal of IRa, using MAFFT (multiple alignments using fast Fourier transform) (Katoh & Standley, 2013). First, we manually checked for small inversions and removed them from the alignment to avoid false results. The intergenic spacer regions, intronic regions, and protein‐coding regions were extracted from the alignment in Geneious R8.1 (Kearse et al., 2012) and visualized in DnaSP v.6 to determine the nucleotide diversity of each region (Rozas et al., 2017). The rates of transition and transversion substitutions were also determined from the pairwise alignment of MAFFT using *B*. *balsamifera* as a reference genome for *B*. *oxyodonta* and *B*. *tenella* (Katoh & Standley, 2013).

### 2.5 Phylogeny in the subfamily Asteroideae

We retrieved 95 species of the subfamily Asteroideae to include species of all the seven main tribes from the NCBI (Table S1) and used them in the inference of phylogeny along with the three species of *Blumea*. *Sonchus acaulis* Dum. Cours. was also retrieved from the NCBI and used as an out‐group from the subfamily Cichorioideae of the family Asteraceae. The plastome sequence of *Chrysanthemoides incana* (Burm.f.) Norl. was extracted from Sequence Read Archive of NCBI under accession number SRR9119075 by mapping to *Ambrosia trifida* following previous approach (Henriquez et al., 2014) to include it as representative of tribe Calenduleae. The complete plastome of all species, after removal of IRb, was aligned using MAFFT. The phylogeny was inferred based on the alignment of 98,453 sites, after removal of indels, using IQ‐TREE2 and related programs (Hoang et al., 2018; Kalyaanamoorthy et al., 2017; Nguyen et al., 2015). The IQ‐TREE2 analyses were based on searching for the best maximum likelihood (ML) tree under the edge‐unlinked partition model (Lopez et al., 2002) and a nonpartitioned dataset with general heterogeneous evolution on a single topology (GHOST) (Crotty et al., 2020). Branch supports values were estimated using the ultrafast bootstrap approximation approach with 10,000 bootstrap replicates (Hoang et al., 2018). The tree structure was drawn and improved by using Interactive Tree Of Life version 4 (Letunic & Bork, 2019).

## 3 RESULTS

### 3.1 Correction of annotations

The initial annotations of plastomes of the subfamily Asteroideae showed variations in both gene and intron content. The plastomes of all species showed similar features after the reannotation of all 26 species of the subfamily Asteroideae. We found various types of errors in annotations related to extra annotations of some genes, and missing annotations for tRNA, protein‐coding genes, introns, and pseudogenes. The most common errors were linked to the genes which contained introns (Table S2).

### 3.2 Plastome organization and features of the genus *Blumea* and the subfamily Asteroideae

**FIGURE 1:**
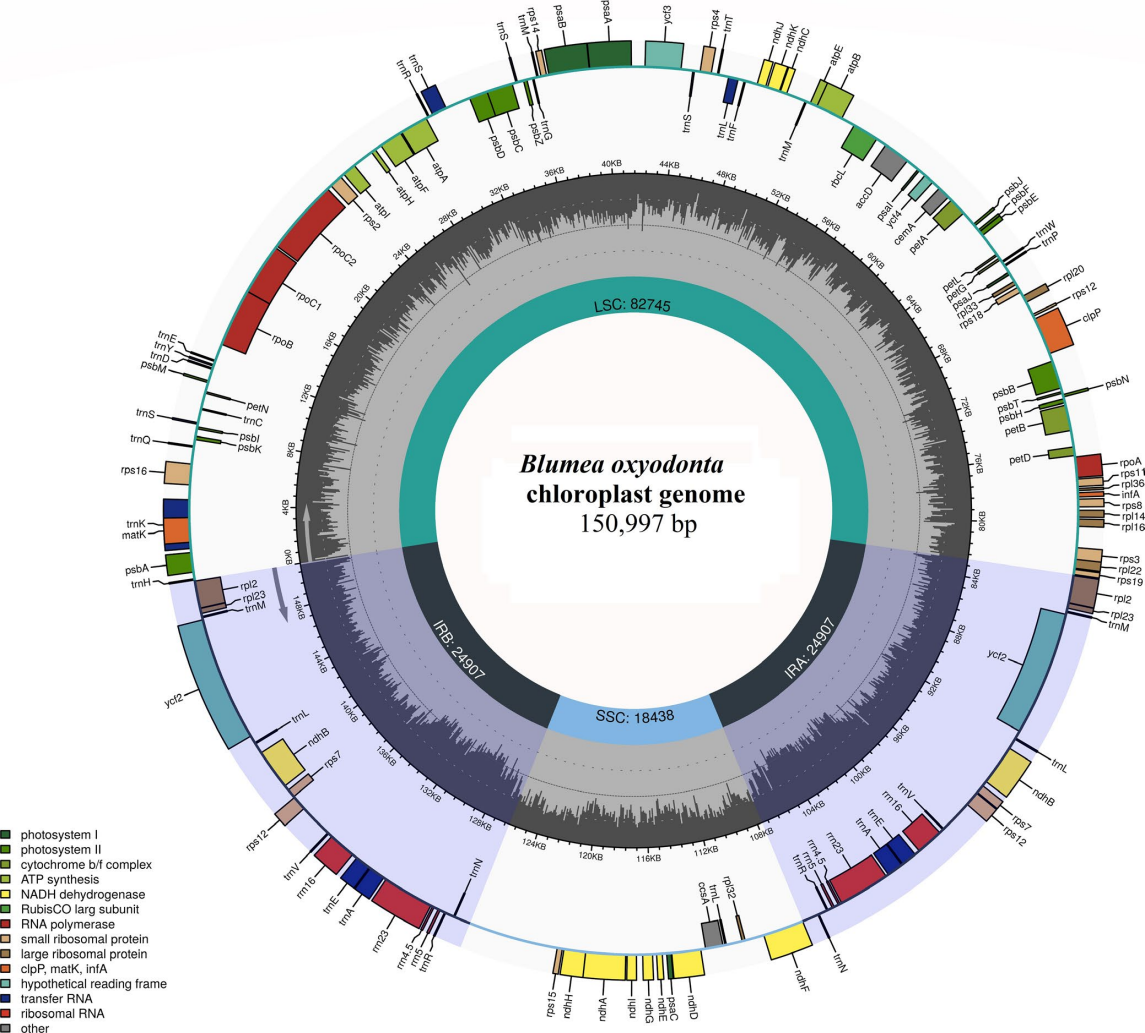
Circular map of plastomes. The color of genes indicates their function. The genes present outside the circle are transcribed counterclockwise, whereas the genes present ​inside the circle are transcribed clockwise. The gene content and organization are similar for all species; therefore, one figure was drawn as representative of all three species

The plastomes were obtained with high average coverage depth ranging from 98× to 130× (Table 1). The three genomes of *Blumea* showed high similarities in genome size. The size of complete plastomes ranged from 150,829 bp (*B*. *tenella*) to 151,176 bp (*B*. *balsamifera*), LSC from 82,478 bp (*B*. *tenella*) to 82,746 bp (*B*. *balsamifera*), SSC from 18,438 bp (*B*. *oxyodonta*) to 18,466 bp (*B*. *balsamifera*), and IR from 24,907 bp (*B*. *oxyodonta*) to 24,982 bp (*B*. *balsamifera*) (Table 2). The plastome of every species contained 113 unique genes, including 29 tRNA, 4 ribosomal RNA (rRNA), and 80 protein‐coding genes. We found 18 duplicated genes in the IR, including 7 tRNA, 4 rRNA, and 7 protein‐coding genes (including *rps12*, a trans‐spliced gene). The guanine‐cytosine (GC) content showed high similarities among the three species throughout the plastome structure and genetic features (Table 2). The *ycf1* gene also left a truncated copy at the junction of IR/SSC along with a functional copy. The genetic organization of the plastomes in *Blumea* species was shown as a circular map using Chloroplot (Figure 1).

**FIGURE 2:**
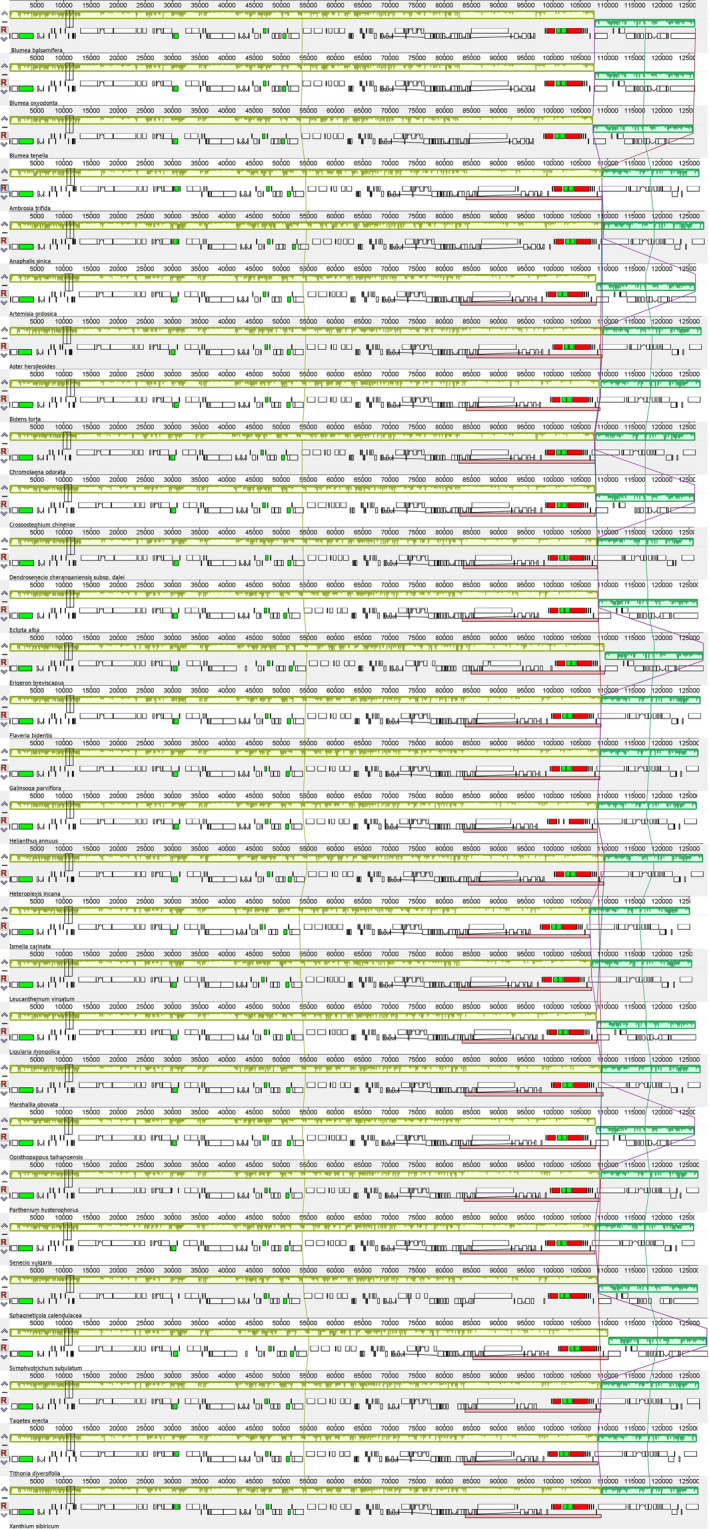
Mauve alignment represents organization of the plastome based on collinear blocks. The figure represents high similarity in all 29 plastomes of Asteroideae while the inversion of the small single copy is also visible from the green block. The small blocks of various colors represent genes. Black = transfer RNA (tRNA); red = ribosomal RNA; white = protein‐coding; green = intron‐containing tRNA

The species of Asteroideae included in the analysis showed similar genetic features and the same number of unique genes. However, duplication of *trnF‐GAA* was detected in *Xanthium sibiricum*. The pseudogene of *rps19* was not observed in the species of *Blumea*, whereas other species of the subfamily Asteroideae also carry a pseudogene along with a functional copy of *rps19* at the junction of IRa/LSC. Mauve‐based analysis showed similarity in gene arrangement and gene content. The rearrangement of genes was not observed in the 29 analyzed species of Asteroideae (Figure 2). The size of complete plastomes ranged from 149,752 bp (*Ismelia carinata* (Schousb.) Sch. Bip.) to 153,318 bp (*Symphyotrichum subulatum*), LSC from 82,290 bp (*I. carinata* (Schousb.) Sch. Bip.) to 85,238 bp (*S. subulatum*), SSC from 17,768 bp (*Dendrosenecio cheranganiensis* (Cotton & Blakelock) E.B. Knox) to 18,488 bp (*Anaphalis* *sinica* Hance), and IR from 24,522 bp (*Leucanthemum virgatum* (Desr.) Clos) to 25,413 bp (*Marshallia obovata*) (Table 2). In addition, the average GC content of the complete plastome was found to be 37%–37.7%, of LSC 34.8%–35.8%, of SSC 30.4%–35.5%, and of IRs 42.7%–43.2%.

**FIGURE 3:**
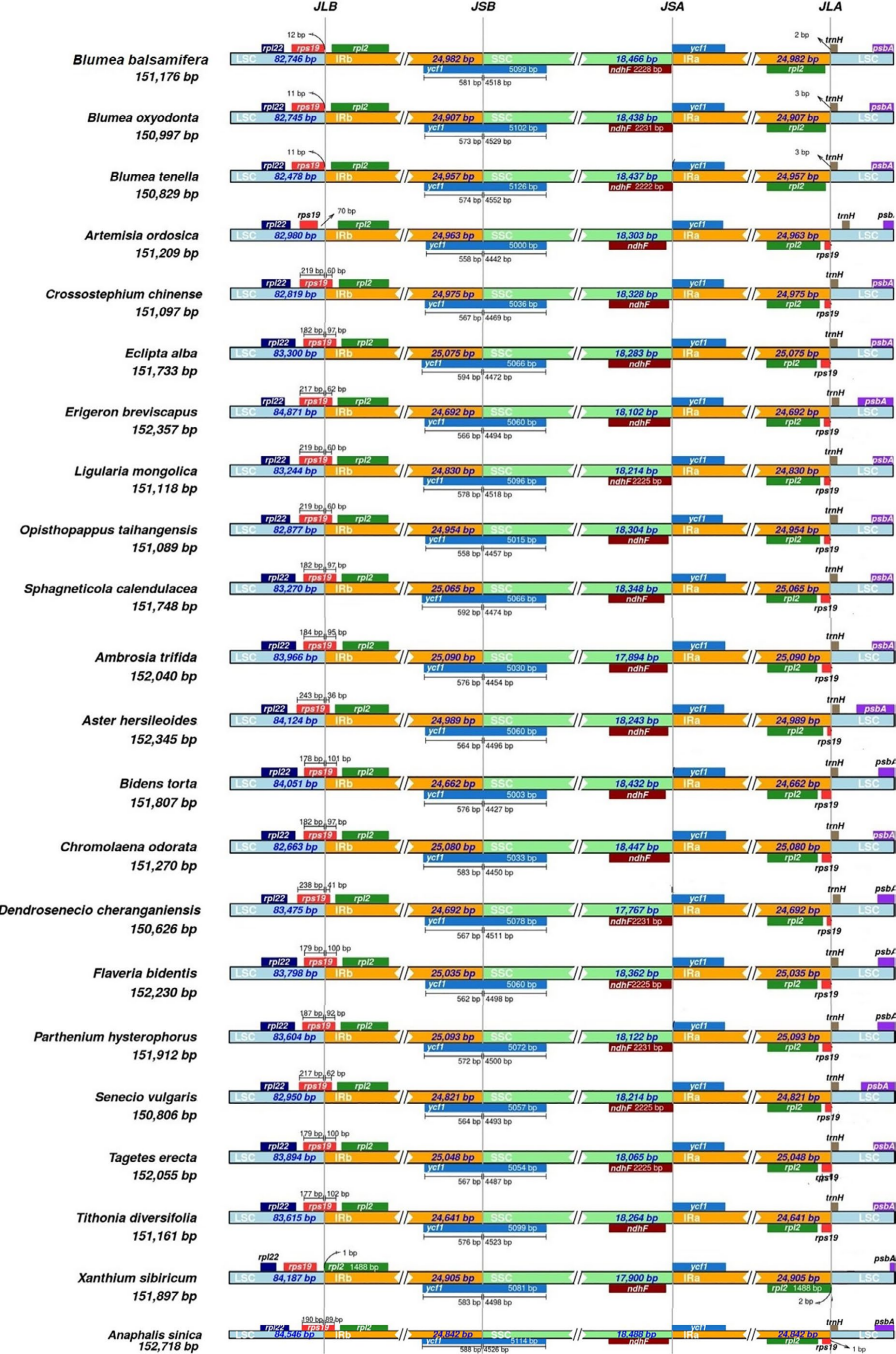
Contraction and expansion of inverted repeats at the junction of plastomes. JLB: LSC/IRb, JSB: IRb/SSC, JSA: SSC/IRa, JLA: IRa/LSC. The genes present above the line transcribe on a negative strand, whereas the gene present below transcribe on a positive strand

Comparative analysis of LSC/IR and SSC/IR junctions showed high similarities in the *Blumea* species and the species of the subfamily Asteroideae (Figure 3). At the LSC/IRb junction (JLB), *rps19* exists entirely in the LSC region in the *Blumea* species and *X. sibiricum*, whereas in all other species *rps19* starts from the IRb and enters LSC leaving a pseudo‐copy of the *rps19* gene at JLA (IRa/LSC). The *rpl*2 gene exists completely in the IR regions away from JLB and JLA. At the SSC/IRa junction (JSA), *ndh*F exists entirely in the SSC region, whereas *ycf*1 started in IR and ended in SSC, as shown at the JSBA (IRb/SSC) and JSA junctions. Hence, a pseudogene of *ycf*1 remains at JSA. At the junction of JLA, the *trn*H gene was found in all species (Figure 3). These data revealed a high resemblance of IR expansion and contraction among species of the subfamily Asteroideae.

### 3.3 Relative synonymous codon usage and amino acid frequency

RSCU and amino acid frequency revealed high similarities among species of *Blumea*. RSCU analysis showed high encoding efficacy of the codon that contained A/T at 3′ with an RSCU ≥1 compared with codons ending with C/G at 3′ with an RSCU <1 (Table S3). We found leucine to be the most frequent amino acid, whereas cysteine was the rarest (Table S4).

### 3.4 Analysis of substitutions and indels

We analyzed the substitution types of the complete plastome and the extent of substitutions and indels in three central plastome regions. We observed a great extent of transversion substitutions relative to transition substitutions and found a transition‐to‐transversion substitution ratio of 0.84–0.87 (Table 3). Most of the substitutions were found in the LSC, followed by SSC and IR (Table 3). Similar distributions were observed for indels. Most indels existed in LSC, followed by SSC and IR (Table 4).

**TABLE 3:** Comparison of substitutions in *Blumea* species

SNPs	*Blumea oxyodonta*	*Blumea tenella*
A/C	243	233
C/T	306	288
A/G	298	280
A/T	121	118
C/G	88	86
G/T	245	238
Total	1,301	1,243
Ts/Tv	0.87	0.84
Distribution of SNPs (by location)		
LSC	944	905
SSC	289	279
IR	68	59

Note

*B. balsamifera* was used as reference for *B. oxyodonta* and *B. tenella*.

Abbreviations: IR, inverted repeat; LSC, large single copy; SNP, single‐nucleotide polymorphism; SSC, small single copy; Ts/Tv, transition‐to‐transversion substitution ratio.

**TABLE 4:** Distribution of indels in *Blumea* plastome

	*Blumea oxyodonta*	Indel length (bp)	Indel average length
LSC	156	956	6.13
SSC	30	174	5.80
IR	13	93	7.15

	*Blumea tenella*	Indel length (bp)	Indel average length
LSC	154	885	5.94
SSC	23	141	6.13
IR	9	49	5.44

Note

*B. balsamifera* was used as reference for *B. oxyodonta* and *B. tenella*.

Abbreviations: IR, inverted repeat; LSC, large single copy; SSC, small single copy.

### 3.5 Analysis of microsatellites and oligonucleotide repeats

**FIGURE 4:**
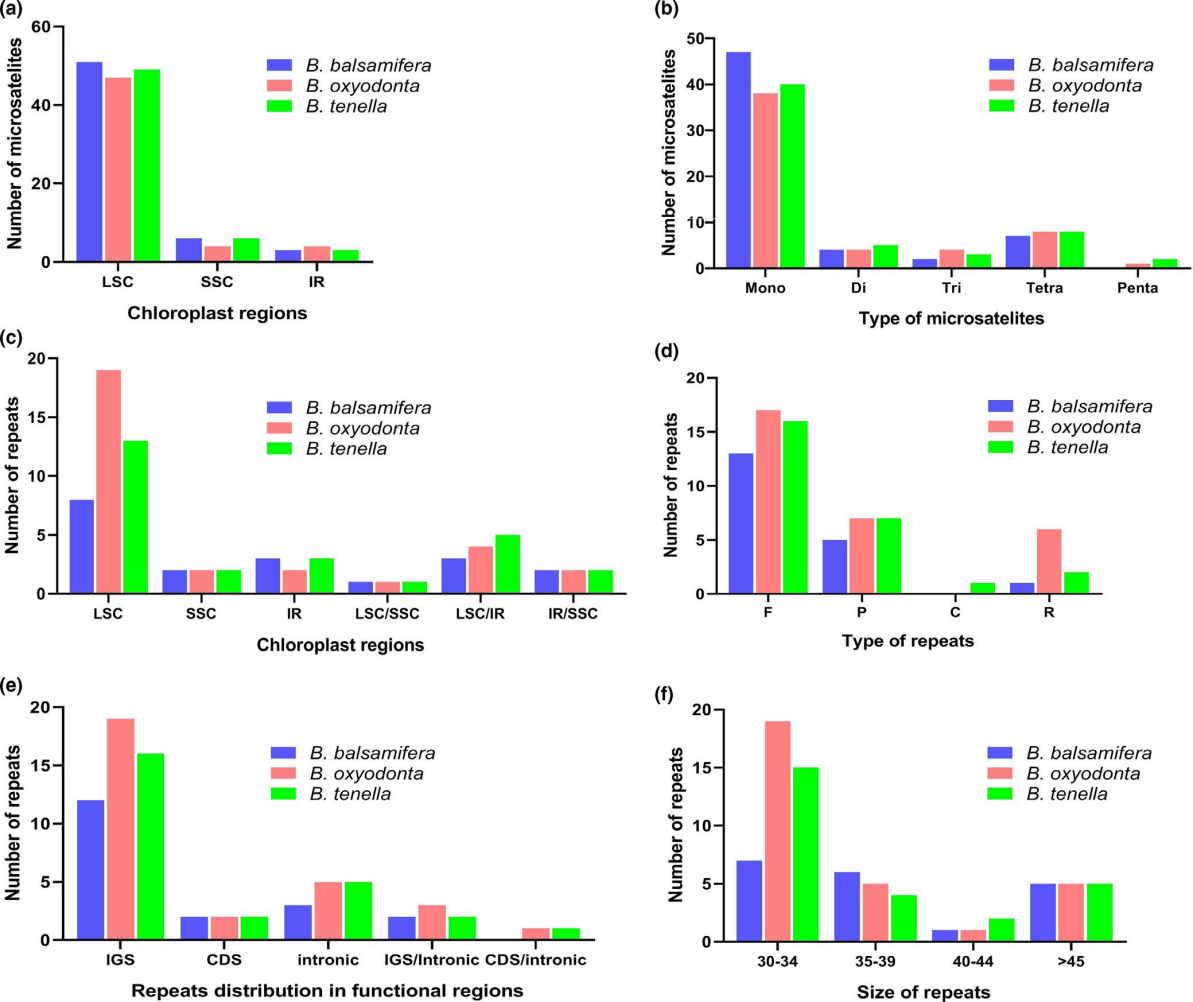
Microsatellite and oligonucleotide repeat analyses. (a) Distribution of microsatellite repeats in three main regions of the plastome. (b) Comparison of the various types of microsatellite repeats. (c) Distribution of oligonucleotide repeats in three main regions of the plastome. (d) Comparison of the various types of oligonucleotide repeats. (e) Distribution of repeats in functional regions of the plastome. (f) Comparison of repeats based on size. IR, inverted repeat; LSC, large single copy; SSC, small single copy; LSC/IR, LSC/SSC, and IR/SSC show those repeats for which one copy of the repeat exists in one region and a second copy exists in another region. C = complementary; CDS = protein‐coding sequence; F = forward repeat; IGS = intergenic spacer region; P = palindromic; R = reverse

We found 55–60 microsatellites among three *Blumea* species. Most of the microsatellites were made up of A/T motifs. Most of the repeats were located in LSC instead of IR and SSC regions (Figure 4a). The mononucleotide microsatellites were most abundant, followed by tetranucleotides (Figure 4b, Table S5). The analysis of oligonucleotide repeats by REPuter detected 75 oligonucleotide repeats in all three species. The highest number of repeats was found in *B*. *oxyodonta* (30), followed by *B*. *tenella* (26) and *B*. *balsamifera* (19). Most of the repeats were present in LSC, followed by IR, regions. Moreover, some repeats were also shared between the LSC, SSC, and IR regions (Figure 4c). Forward repeats showed an abundance relative to other types of repeats (Figure 4d). The intergenic spacer regions contained more repeats than the intronic and coding regions (Figure 4e). Most of the repeats were between 30 bp and 34 bp in size (Figure 4f). Details about oligonucleotide repeats are provided in Table S6.

### 3.6 Identification of polymorphic loci

**FIGURE 5:**
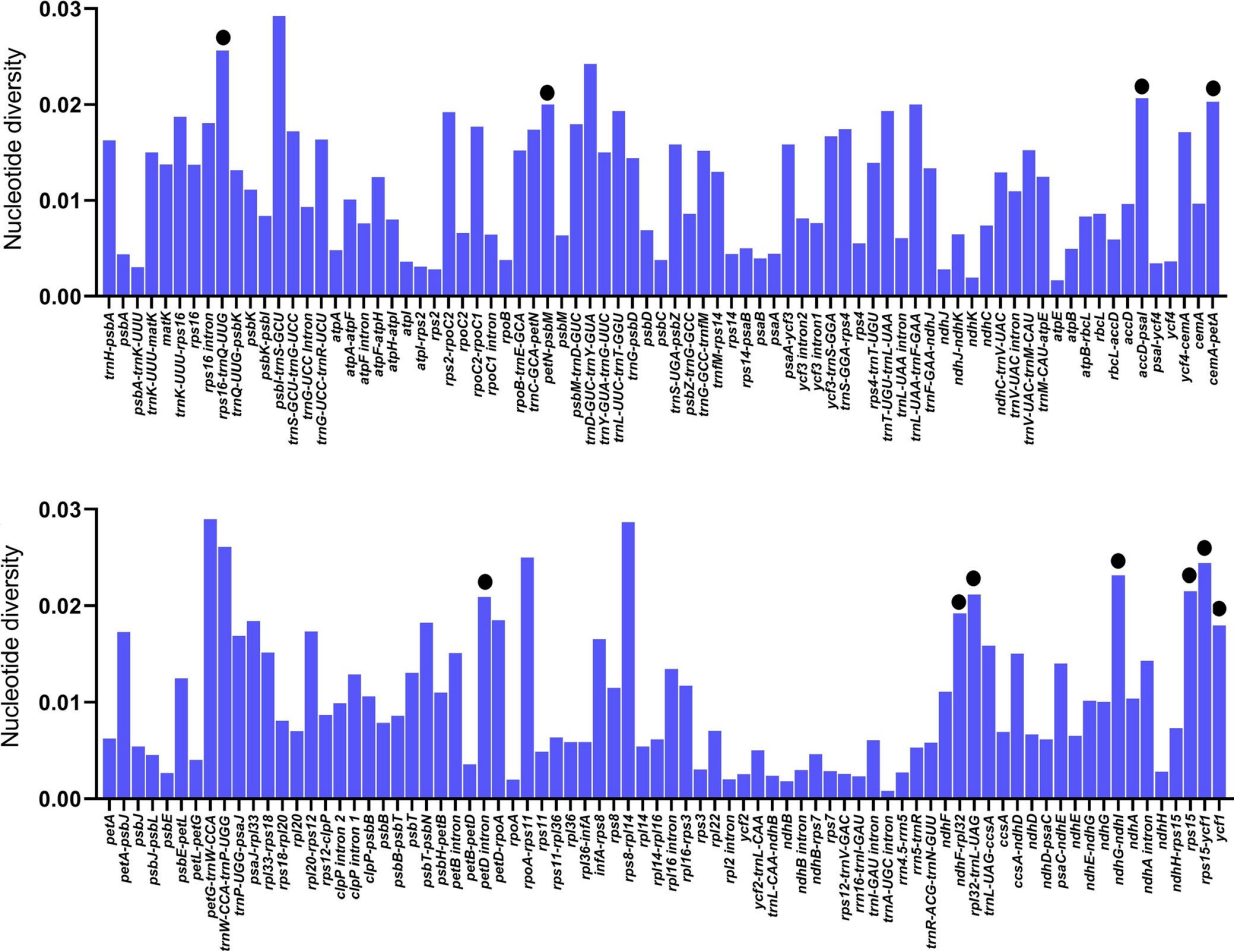
Extent of polymorphism in all plastid regions. Regions with no nucleotide diversity were excluded and are not shown here. The black circle indicates the 10 suitable polymorphic loci with length >200. The *x*‐axis shows plastid regions and the *y*‐axis nucleotide diversity

We recorded the highest average polymorphism for intergenic spacer regions (0.0121) as compared with intronic regions (0.0096) or protein‐coding sequences (0.0047). The polymorphism of all regions is shown in Figure 5. We ignored loci <200 bp and selected 10 polymorphic regions with nucleotide diversity >0.02, of which 6 belonged to intergenic spacer regions, 1 to intronic, 1 to protein‐coding, and 1 to both intergenic spacer and coding regions. The 730 bp region of *ycf*1 was selected instead of the complete gene using the oligonucleotide repeat as a proxy. The chosen part showed a nucleotide diversity of 0.0252 and contained 28 substitutions events with zero missing data. A similar approach was used for *ndh*F*‐rpl*32, selecting an 841 bp region, which had a nucleotide diversity of 0.0206 and contained 26 substitutions. The selected regions may act as suitable and cost‐effective markers (Table 5).

**TABLE 5:** Identified suitable polymorphic loci based on comparative plastome analysis of *Blumea* species

Serial number	Region	Nucleotide diversity	Number of substitutions	Number of indels	Region length	Alignment length	Missing data (%)
1	*rps*16‐*trn*Q*‐UUG*	0.02561	34	5	894	936	4.49
2	*ycf*1	0.02557	28	0	730	730	0
3	*rps*15 and *rps*15‐*ycf*1	0.02516	24	4	636	686	7.28
4	*ndhG‐ndhI*	0.02313	11	5	317	356	10.9
5	*rpl*32‐*trnL‐UAG*	0.02119	28	3	865	887	2.48
6	*pet*D intron	0.02092	22	1	701	711	1.4
7	*acc*D‐*psa*I	0.02066	23	5	726	760	4.68
8	*ndh*F‐*rpl*32	0.02061	26	4	841	850	1.06
9	*cem*A‐*pet*A	0.02029	7	0	230	230	0
10	*pet*N‐*psb*M	0.02004	15	4	499	510	2.16

Abbreviation: Indel, Insertions/deletions.

### 3.7 Phylogenetic inference of the species of the genus *Blumea* with 95 other species

**FIGURE 6:**
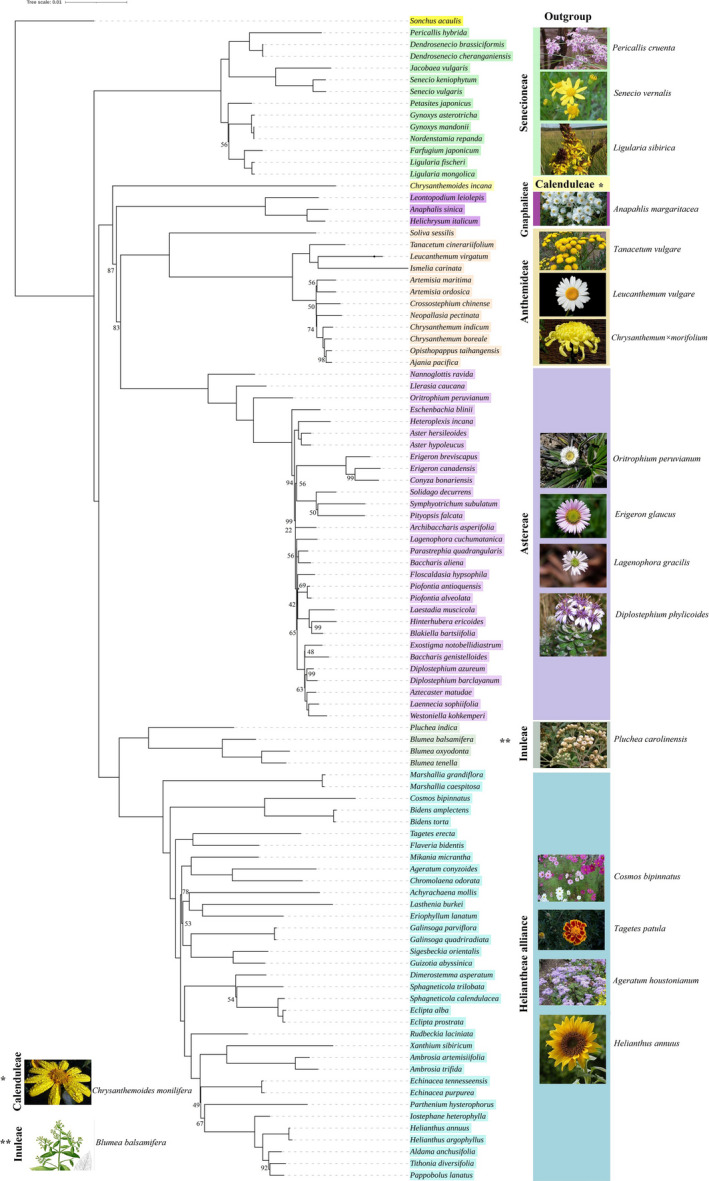
Phylogenetic inference among 98 species belonging to 7 tribes of the subfamily Asteroideae using *Sonchus acaulis* as out‐group. Species of each tribe are shown by different color for clarity. The bootstrapping value equal to 100 is omitted from each node and is not shown

The species of the genus *Blumea* lay on the same node and share a node with a high bootstrapping support of 100 with *Pluchea indica*. Our result was based on sequences of the complete plastome and showed the placement of *Blumea* in the tribe Inuleae. Moreover, the phylogenetic relationship was also described between the seven tribes of the subfamily Asteroideae (Figure 6). Our phylogenetic inference showed that the Heliantheae alliance is the most recently diverged tribe of the subfamily Asteroideae, which forms a common node with the tribe Inuleae. The tribe Astereae was closely related to Anthemideae, whereas Gnaphalieae forms the first branching node of these two tribes. The tribe Calenduleae roots Gnaphalieae, which is rooted finally by Senecioneae. Hence, the species of the tribe Senecioneae lie in the first branching node of the subfamily Asteroideae in our phylogeny.

## 4 DISCUSSION

We de novo assembled the plastomes of three *Blumea* species and compared them with 26 other Asteroideae species. We provided insight into plastome structure, IR contraction and expansion, and suitable polymorphic loci.

### 4.1 Plastome comparison of *Blumea* and Asteroideae

The plastome of the three *Blumea* species and the other 26 species of the subfamily Asteroideae showed high similarities in genome structure, gene organization, and genetic content without any inversion linked to the rearrangement of genes. Previous studies of angiosperms have also shown conserved plastomes in various plant lineages such as Solanaceae, Malvaceae, and Araceae in which the same gene content and gene order were previously reported (Abdullah, Henriquez, Mehmood, et al., 2020; Abdullah, Henriquez, Mehmood, Shahzadi, et al., 2020; Abdullah, Mehmood, et al., 2020; Amiryousefi et al., 2018a; Mehmood, Abdullah, Shahzadi, et al., 2020). The slow rate of evolution and typically conservative nature of the plastome is linked to various molecular mechanisms such as the organization of plastid genes in operons, uniparental inheritance (maternal or paternal), the presence of an active repair mechanism, and rarity of plastid fusion (Wicke et al., 2011). Nevertheless, a rearranged plastome and loss of certain genes are reported in various plant lineages such as the ferns, Pinaceae, and Cyperaceae (Daniell et al., 2016; Lee et al., 2020; Poczai & Hyvönen, 2011; Wicke et al., 2011). Hence, the plastomes of Asteroideae avoid the rearrangements by a certain mechanism. The GC content of the plastomes was also similar to previous Asteraceae genomes and of other plant lineages, and high GC content was observed in IRs, which might be due to the presence of rRNAs, as they have a GC content of up to 55% (Amiryousefi et al., 2018a; Jung et al., 2021; Kim et al., 2019; Lin et al., 2019; Poczai & Hyvönen, 2017).

We found various types of errors in the annotations of 26 species of Asteroideae. This shows that the reported genomes have certain errors in annotations as stated previously in a detailed study of the family Solanaceae (Amiryousefi et al., 2018a) and the comparative genomics of the two species of Malvaceae (Abdullah, Waseem, et al., 2020). The gene content was found to be the same in all the species of Asteroideae after correction of annotations, which is also in agreement with gene features in the previously reported plastome of other subfamilies such as Cichorioideae, Pertyoideae, and Carduoideae (Jung et al., 2021; Kim et al., 2019; Lin et al., 2019). Hence, these data showed the highly conservative plastomes of Asteraceae. If we considered the previously reported annotations accurately without repeating the annotations, large variations were evident in the analyzed species. Hence, based on these observations, together with a previous report on the Solanaceae family (Amiryousefi et al., 2018a), we suggest a correction of annotations before comparative genomics to ensure accurate data regarding the plastome structure, genetic content, intron content, and for various other analyses related to the study of plastome evolution.

The expansion and contraction of IRs showed much similarity among the species of the subfamily Asteroideae. This result agrees with previous studies of other angiosperms such as Malvaceae (Abdullah, Mehmood, et al., 2020) and Solanaceae (Amiryousefi et al., 2018a). The pseudogene of *ycf*1 originated at the junction of IR. The origination of the pseudogene due to IR expansion and contraction is expected and observed in other angiosperms (Abdullah, Henriquez, Mehmood, Carlsen, et al., 2020; Iram et al., 2019; Mehmood, Abdullah, Ubaid, et al., 2020). Previous studies have shown a resemblance at the junctions of the plastome in closely related species (Liu et al., 2018). A similar phenomenon can be seen in the closely related species of *Blumea* and the subfamily Asteroideae. Detailed studies of the family Araceae do not support this suggestion, however, instead showing a high level of variation at junctions of the plastome even in closely related species (Abdullah, Henriquez, Mehmood, Carlsen, et al., 2020; Abdullah, Henriquez, Mehmood, Shahzadi, et al., 2020). The expansion and contraction of IRs also led to duplication of single‐copy genes (genes that travel from SSC or LSC to IRs become duplicated) or conversion of otherwise duplicated genes to single‐copy genes (genes that move from IRs to LSC or SSC become single copy) (Zhu et al., 2016). This traveling of the gene also affects the rate of mutations; mostly, the genes that travel from LSC or SSC to IRs showed a low rate of evolution or vice versa (Abdullah, Henriquez, Mehmood, Carlsen, et al., 2020; Zhu et al., 2016). The high similarities in junctions also indicate the presence of the same genes in all the species, and the total number of genes does not vary due to IR expansion and contraction.

### 4.2 Repeat analysis and utilization of oligonucleotide repeats as proxy to identify polymorphic loci

Microsatellites are very important for the study of population genetics. Besides hexanucleotide, we detected mononucleotide, dinucleotide, trinucleotide, tetranucleotide, and pentanucleotide microsatellites in which mononucleotide repeats were abundant, followed by tetranucleotide repeats. A similar pattern of repeats was observed in other Asteraceae (Sablok et al., 2019). Most of the nucleotides were made up of A/T motifs instead of C/G motifs. This might be due to the A/T‐rich plastome structure, as observed in other angiosperms (Iram et al., 2019; Mehmood, Abdullah, Shahzadi, et al., 2020). The identified microsatellites in the current study may be helpful in population genetic studies of *Blumea*.

Oligonucleotide repeats exist widely in the plastome (Abdullah, Mehmood, et al., 2021; Ahmed et al., 2012, 2013). These repeats play a role in generating mutations and have been suggested as a proxy to identify mutational hotspots (Abdullah, Mehmood, et al., 2020, 2021; Ahmed et al., 2012). Abdullah, Mehmood, et al. (2020) recently reported the co‐occurrence of up to 90% of repeats with substitutions, whereas 36%–91% co‐occurrence was recorded at the genus level. In the current study, we identified 10 highly polymorphic loci. Among these, five loci belong to the regions where repeats are present, including *rps*16‐*trn*Q and *ycf*1, which showed the highest incidence of polymorphisms. Here, our findings support the use of repeats as a proxy, and this approach may also be helpful for the identification of suitable polymorphic loci for phylogenetic inference of other taxonomically complex genera. This approach is promising since the plastome of a single species can be used to identify polymorphic regions. Repeated coding regions and IR regions need to be avoided, however, due to the purifying selection pressure of protein‐coding genes (Henriquez et al., 2020b) and the fact that copy‐dependent repair mechanisms (Zhu et al., 2016) lead to low rates of mutation.

### 4.3 Suitable polymorphic loci for resolving phylogenetic discrepancies of *Blumea*

Regions of the plastome showed different polymorphisms, and certain regions are more predisposed to mutations (Abdullah, Mehmood, et al., 2020; Henriquez et al., 2020b; Mehmood, Abdullah, Ubaid, et al., 2020; Shahzadi et al., 2020). All regions are therefore not equally important for phylogenetic inference and barcoding of plant species (Daniell et al., 2016; Li et al., 2014). Pornpongrungrueng et al. (2007) used *trn*H‐*psb*A and *trn*L‐F of plastome along with ITS1 and ITS2 from the nuclear genome to resolve the phylogeny of *Blumea*. The regions of plastome showed very low polymorphism. The ITS region showed speedy evolution and its sequences generated a lot of missing data when aligned due to a high rate of insertion and deletion events. Hence, the phylogenetic tree drawn for the ITS region and concatenated sequences of plastome and ITS regions cannot resolve interspecific relationships and showed low bootstrapping support for various nodes. Hence, the authors suggested the use of highly polymorphic markers with broad sampling to resolve the phylogeny. Later, Pornpongrungrueng et al. (2009) again attempted to resolve the phylogeny of *Blumea*. They also validated the 5S‐NTS region from the nuclear genome along with *trn*H‐*psb*H, *trn*L‐F, and ITS. The 5S‐NTS showed speedy evolution and performed worse than the ITS and chloroplast regions. According to the authors, several distinct paralogous sequences within an individual may be the main reason for the low phylogenetic signal of 5S‐NTS. The authors also could not resolve the phylogeny of several species, such as *Blumea lacera* (Burm.f.) DC., *B*. *oxyodonta*, *Blumea megacephala* (Randeria) C.T. Chang & C.H. Yu ex Y. Ling, *Blumea mollis* (D. Don) Merr., and *Blumea saxatilis Zoll*. *ex* *Zoll. & Moritzi*. Moreover, at the species level, several relationships were observed with low bootstrap / without bootstrap support. Hence, to resolve the phylogeny, the identification of suitable polymorphic loci was suggested by the authors. The complete plastome was suggested for barcoding and phylogenetic inference (Li et al., 2014), but the high cost hinders its use. Therefore, identifying species‐specific suitable polymorphic loci can provide a quality resource for the phylogenetic inference and barcoding of plant species (Ahmed et al., 2013; Li et al., 2014). Here, we identified 10 polymorphic loci that are different and more polymorphic than previously employed loci (*trn*H‐*psb*A and *trn*L‐F) (Pornpongrungrueng et al., 2007; Zhang et al., 2019) of the plastome. Our identified loci also included intergenic spacer regions along with coding and intronic regions. Our approach agrees with recent studies in which intergenic spacer regions were also suggested at the low taxonomic level for phylogenetic inference in Bignonieae of the family Bignoniaceae and the genus *Artemisia* of Asteraceae (Shahzadi et al., 2020; Thode et al., 2020). Our identified polymorphic loci showed low polymorphism compared with ITS regions. Still, the generation of no/low missing data of our identified polymorphic loci relative to the ITS makes these loci appropriate for phylogenetic inference and barcoding of *Blumea* species. Moreover, the ITS regions show low amplification success during polymerase chain reaction and possible contamination with fungus sequences, rendering the downstream processes costly and time‐consuming (Li et al., 2014). The species‐specific markers of the plastome show high amplification success and are not contaminated with fungus sequences, being found to be authentic, robust, and cost‐effective in recent studies (Abdullah, Henriquez, Croat, et al., 2021; Abdullah, Henriquez, Mehmood, et al., 2021; Ahmed, 2014; Ahmed et al., 2013; Li et al., 2014; Nguyen et al., 2018). Hence, our identified polymorphic loci may also be authentic, robust, and cost‐effective for the barcoding and phylogenetic inference of the genus *Blumea*.

### 4.4 Conflicting signals in the phylogeny of Asteroideae

Phylogenetic analysis of 98 species of the subfamily Asteroideae based on the complete plastome shows that the genus *Blumea* lies in the tribe Inuleae and is closely related to the genus *Pluchea*. The same relationship was previously observed based on the *ndh*F gene of plastid (Anderberg et al., 2005). The tribal‐level phylogenetic relationship of our study is similar to the previous studies of the subfamily Asteroideae reported based on plastome sequences (Fu et al., 2016; Panero & Crozier, 2016). However, similar to previous studies, the phylogenetic analysis in our recent study was based on the complete plastome, which also conflicts with the phylogenetic inference of the family performed based on the nuclear genome (Mandel et al., 2019). Mandel et al. (2019) stated a sister relationship between “Senecioneae and Anthemideae” and “Astereae and Gnaphalieae.” However, our study and previous reports (Fu et al., 2016; Panero & Crozier, 2016; Watson et al., 2020) based on plastome data show the sister relationship between “Astereae and Anthemideae,” while Gnaphalieae roots these two tribes which is rooted by Calenduleae, whereas Senecioneae presents at the base of Asteroideae. A recent phylogeny based on nuclear data showed a sister relationship between Astereae and Gnaphalieae rooted as Calenduleae, Anthemideae, and then Senecioneae (Watson et al., 2020). These results of nuclear phylogeny are also similar to the previous report (Huang et al., 2016) in which authors showed the same relationships based on transcriptomic data. However, they have not included the species of Anthemideae. Watson et al. (2020) demonstrated evidence for four reticulate events: three intratribal (within Astereae, Anthemideae, and Senecioneae) and one intertribal (between Anthemideae and Gnaphalieae). They suggested that ancient reticulating events within the five tribes may be possible, which further confounds any conclusion about the existing evolutionary history of these five tribes. Asteroideae diverged recently (~37 mya) (Mandel et al., 2019) during the Mid‐Eocene Climatic Optimum, when the temperature of the globe decreased continuously, or somewhat earlier (~45 mya) (Panero & Crozier, 2016). Hence, the accelerated rate of diversification may be responsible for the conflicting signals due to the loss of some of the earliest lineages of the Asteroideae (Watson et al., 2020). Vargas et al. (2017) also observed conflicting signals for the data set of nuclear, plastome, and mitochondrial genomes in *Diplostephium* and in aligned genera of Astereae. They provided evidence for reticulate evolution in events of rapid diversification in the analyzed species of Astereae and suggested that the phylogeny based on plastome and mitochondria sequences contradict with nuclear due to uniparental inheritance of these genomes. In the current study, the conflicting signal among the aforementioned tribes may also be due to reticulate evolution in events of rapid diversification. Moreover, the uniparental inheritance of the plastome may also confound phylogenetic inference, which might require further investigation.

In conclusion, our study provides insight into plastome structure evolution of the genus *Blumea* and the subfamily Asteroideae. The identified polymorphic loci were linked to the location of oligonucleotide repeats and confirm the role of repeats as a proxy for the identification of polymorphic loci. The 10 identified loci may facilitate barcoding and phylogenetic inference of the genus *Blumea*. However, some practical validation may be required of the identified loci. Our study shows the conflicting signals between plastome and nuclear phylogeny at tribal levels, which also requires further investigation.

## CONFLICT OF INTEREST

The authors have no conflicts of interest to declare.

## AUTHOR CONTRIBUTION

**Abdullah:** Conceptualization (equal); Data curation (lead); Formal analysis (lead); Investigation (lead); Project administration (equal); Software (lead); Validation (equal); Writing‐original draft (lead). **Furrukh Mehmood:** Data curation (supporting); Formal analysis (supporting). **Abdur Rahim:** Data curation (supporting); Formal analysis (supporting). **Parviz Heidari:** Data curation (supporting); Formal analysis (supporting); Writing‐review & editing (supporting). **Ibrar Ahmed:** Conceptualization (equal); Investigation (equal); Project administration (supporting); Supervision (equal); Writing‐review & editing (equal). **Péter Poczai:** Conceptualization (equal); Data curation (equal); Methodology (equal); Project administration (equal); Supervision (equal); Visualization (equal); Writing‐review & editing (equal).

## Supporting information

Table S1

Table S2

Table S3

Table S4

Table S5

Table S6

## Data Availability

Plastome sequences of the three species *Blumea oxyodonta* (BK013128), *Blumea tenella* (BK013129), and *Blumea balsamifera* (BK013127) were submitted to GenBank of the National Center for Biotechnology Information. Data of all the other species are available in GenBank and their accession numbers are provided in Tables 1 and S1. The detailed methodology is mentioned in the manuscript, which makes the study reproducible.
